# Binding Ensembles of *p53*-MDM2 Peptide Inhibitors by Combining Bayesian Inference and Atomistic Simulations

**DOI:** 10.3390/molecules26010198

**Published:** 2021-01-02

**Authors:** Lijun Lang, Alberto Perez

**Affiliations:** Chemistry Department, University of Florida, Gainesville, FL 32611, USA; lijunlang@chem.ufl.edu

**Keywords:** IDP 1, binding 2, molecular dynamics 3, MELD×MD 4, advanced sampling 5, *p53* 6, MDM2 7

## Abstract

Designing peptide inhibitors of the *p53*-MDM2 interaction against cancer is of wide interest. Computational modeling and virtual screening are a well established step in the rational design of small molecules. But they face challenges for binding flexible peptide molecules that fold upon binding. We look at the ability of five different peptides, three of which are intrinsically disordered, to bind to MDM2 with a new Bayesian inference approach (MELD × MD). The method is able to capture the folding upon binding mechanism and differentiate binding preferences between the five peptides. Processing the ensembles with statistical mechanics tools depicts the most likely bound conformations and hints at differences in the binding mechanism. Finally, the study shows the importance of capturing two driving forces to binding in this system: the ability of peptides to adopt bound conformations ($\Delta G_{conformation}$) and the interaction between interface residues ($\Delta G_{interaction}$).

## 1. Introduction

Peptide molecule inhibitors have the potential to bind to proteins classified as “undruggable” by small molecules thanks to their flexibility and complementary nature to proteins [1,2]. Rational drug design of small molecules via computational tools (e.g., docking of virtual libraries) is a common practice in the drug discovery process. However, these tools are not well suited to handle the flexible nature of peptide molecules, many of which are intrinsically disordered and only adopt stable structures in the presence of their binding partners [3].

Modeling the binding of flexible molecules continues to be a grand challenge in computational structure prediction. In recent years, with the increase of peptide therapeutics in the market there has been a continuous development and adaptation of docking tools to capture protein-peptide interactions [3,4]. Docking programs address the flexibility of peptides by two main routes: (1) using homology models, PDB (Protein Data Bank)structural motifs, or other sources of structures for docking [5,6,7,8]; and (2) provide peptide flexibility for folding upon binding [9,10,11,12,13,14]. Initial peptide conformations for docking could come from computationally expensive molecular dynamics (MD) simulations of the free peptide. However, many such peptides are intrinsically disordered (IDP), limiting their use [7]. Full exploration of folding upon binding through standard molecular dynamics becomes too computationally demanding [15], requiring advanced sampling strategies to efficiently sample the energy landscape.

In this work, we take a look at binding and free-peptide ensembles (simulating the peptide in isolation) for different peptides to better understand the nature of the *p53*-MDM2 interaction. *p53* is called the guardian of the genome, triggering programmed death (apoptosis) when cells misbehave. MDM2 down-regulates *p53* limiting its tumor suppressor activity. Thus, inhibitors of the *p53*-MDM2 and the closely related MDMX interaction have long been a cancer drug target [16,17,18]. Multiple studies of the native interaction [19,20,21,22] and the ability to design inhibitors that simultaneously block MDM2 and MDMX [23,24,25] provide a wealth of data to assess new computational tools. Since binding simulations are more computationally demanding than free peptide simulations, our goal is to identify peptide properties that might make the peptide a better binder–leading to faster computational screening of peptide therapeutics.

**Figure 1:**
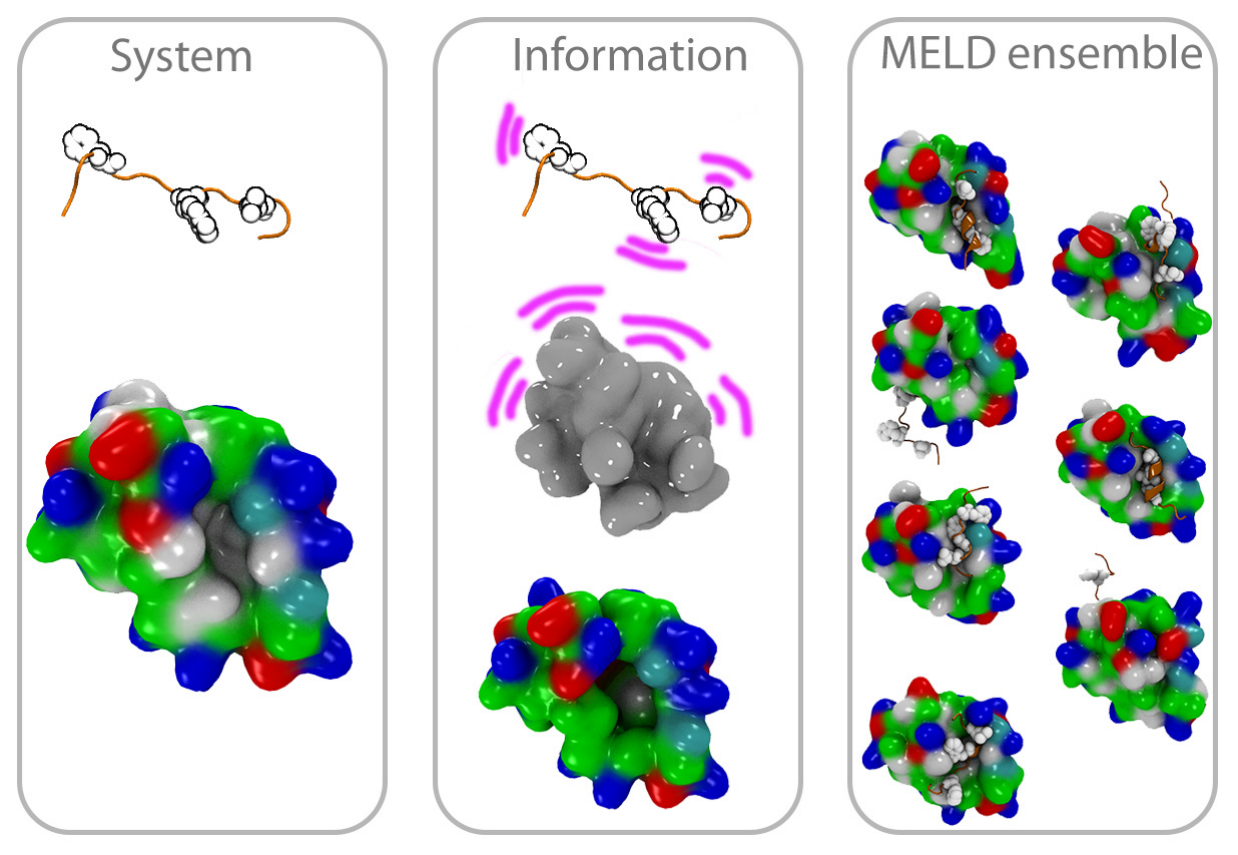
Outline of the MELD×MD setup. We start with the peptide far from MDM2 (system). We use noisy information to favor sampling of binding/unbinding events (middle panel). A statistical mechanics of the posterior distribution coming from the MELD ensemble identifies conformations that are most consistent with the force field and a subset of the data, and we compare these to the experimental structure.

The *p53*-MDM2 interaction is characterized by three hydrophobic residues (Phe19, Trp23 and Leu26) from the peptide which anchor into a deep cavity in MDM2. In order for the three hydrophobic residues to align with the pocket, the *p53* epitope adopts a helical conformation. This is in contrast with the IDP nature of the peptide in isolation. We use noisy information to guide binding using our previously developed Bayesian inference approach (MELD×MD [26]) to identify the subset of data that is most compatible with the force field and the resulting bound conformations (see Figure 1). To further test the methodology, we simulated five different peptides, including the peptide epitope from *p53*, two inhibitors, and two alanine-based peptides that we do not expect to be good binders, as control. The work highlights the ability of molecular dynamics tools to capture the two driving forces behind binding: preferences of the peptides to adopt bound-like conformations and the use of binding simulations to differentiate binding preferences.

## 2. Results

### 2.1. Free Peptide Simulation Ensembles Show the IDP Nature of p53

**Figure 2:**
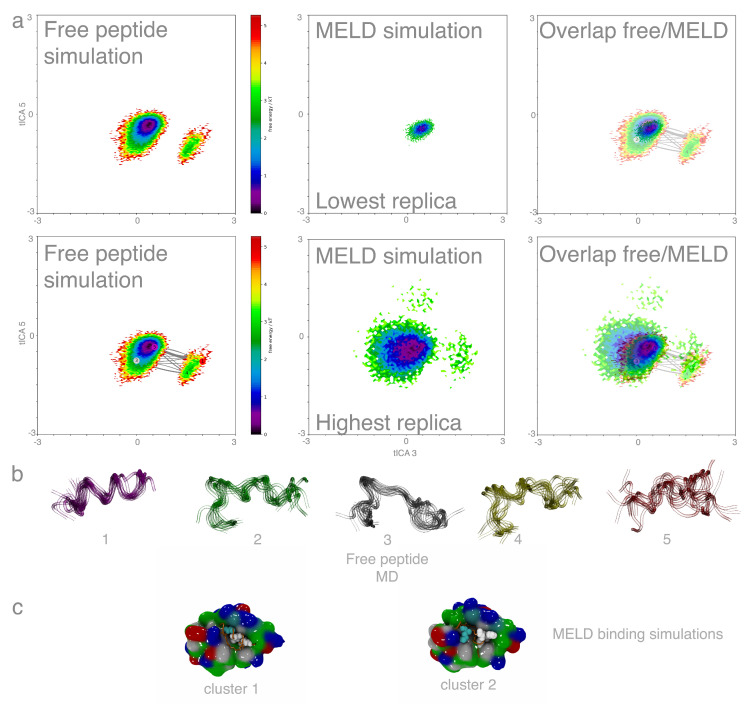
Comparison of the conformational space for free peptide versus binding simulations for the *ATSP*-7041 peptide. (**a**) The peptide ensembles are projected onto the third and fifth tICA eigenvectors common to all five peptides. (**b**) The metastable states sampled for the free peptide. (**c**) Top clusters by population from MELD×MD binding simulations.

**Figure A1:**
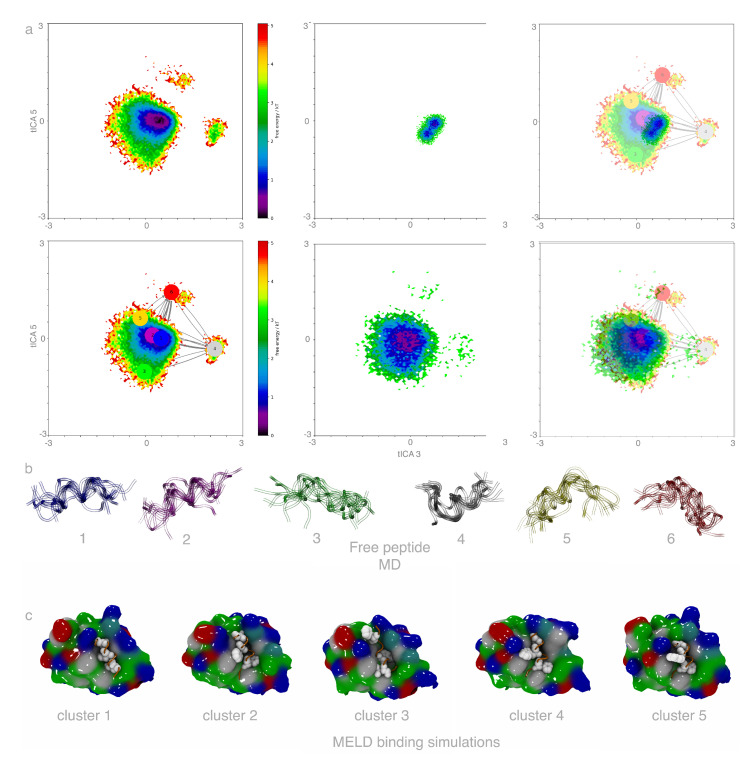
Comparison of the conformational space for free peptide versus binding simulations for the *pdiq* peptide. (**a**) The peptide ensembles are projected onto two tICA eigenvectors common to all five peptides. (**b**) The metastable states sampled for the free peptide. (**c**) Top clusters by population from MELD×MD binding simulations.

**Figure A2:**
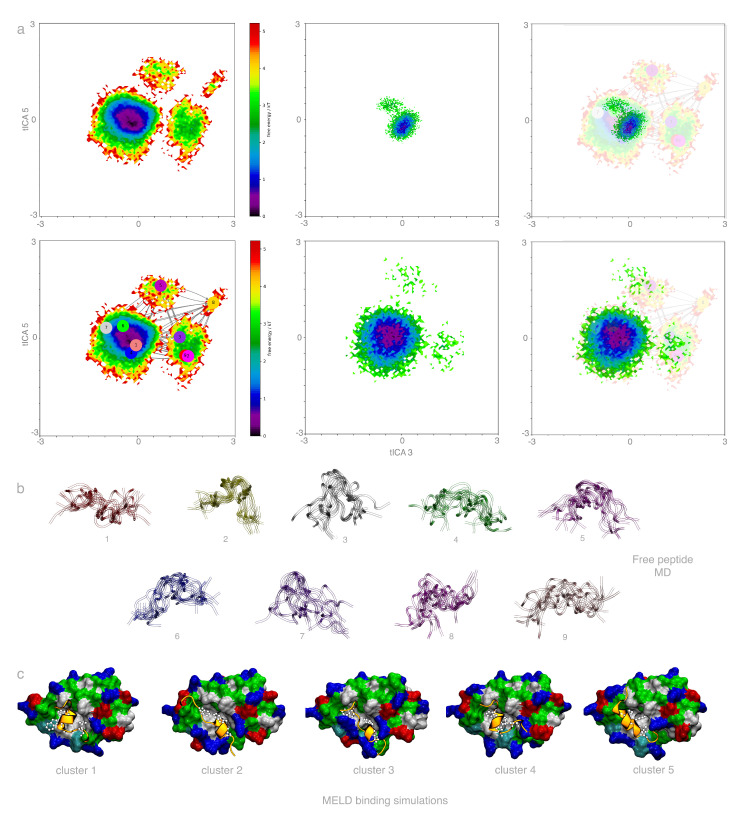
Comparison of the conformational space for free peptide versus binding simulations for the *p53* epitope. (**a**) The peptide ensembles are projected onto two tICA eigenvectors common to all five peptides. (**b**) The metastable states sampled for the free peptide. (**c**) Top clusters by population from MELD×MD binding simulations.

**Figure A3:**
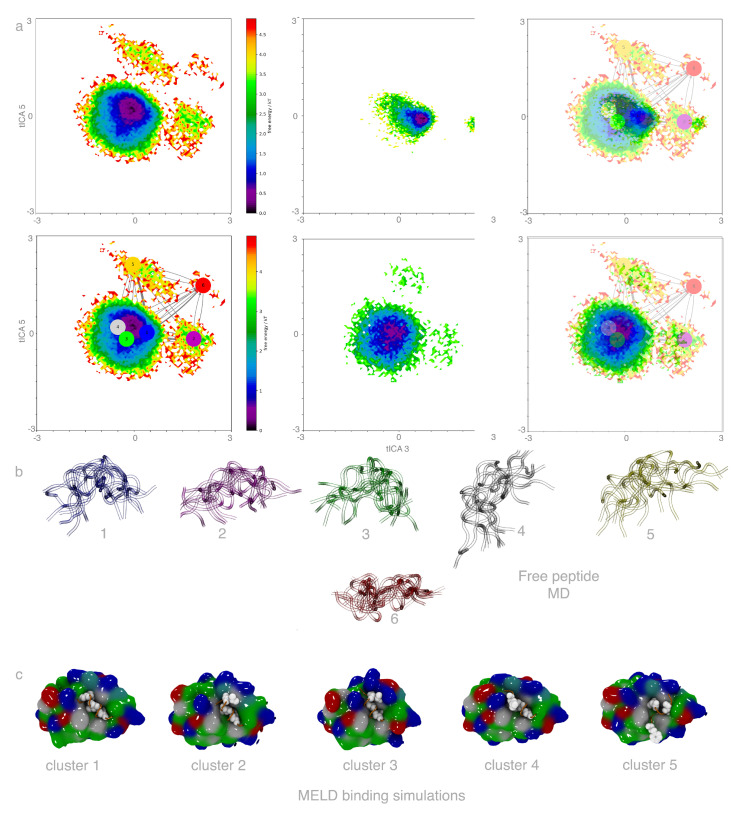
Comparison of the conformational space for free peptide versus binding simulations for *Ala1*. (**a**) The peptide ensembles are projected onto two tICA eigenvectors common to all five peptides. (**b**) The metastable states sampled for the free peptide. (**c**) Top clusters by population from MELD×MD binding simulations.

**Figure A4:**
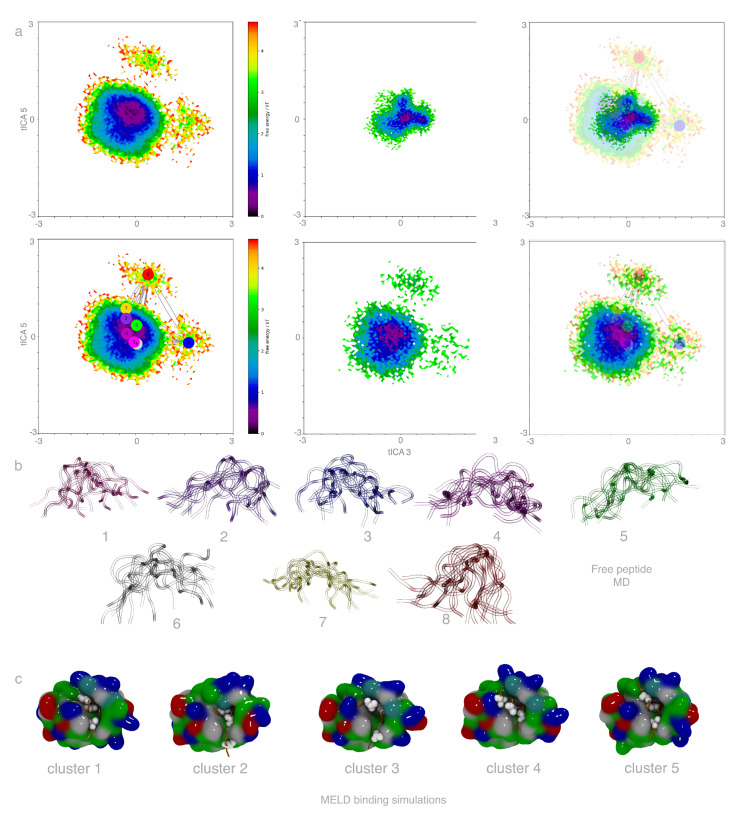
Comparison of the conformational space for free peptide versus binding simulations for *Ala2*. (**a**) The peptide ensembles are projected onto two tICA eigenvectors common to all five peptides. (**b**) The metastable states sampled for the free peptide. (**c**) Top clusters by population from MELD×MD binding simulations.

We simulated five peptides in their free form (see Methods and Table 1), capturing their intrinsic degree of disorder. All peptides are able to visit multiple states with short life times. A 2D-RMSD clustering of the ensemble reveals many clusters with low populations for the *p53* and two control peptides, consistent with their intrinsically disordered nature (see unrestrained Molecular Dynamics (MD) column in Table 2). The peptide pdiq adopts stable helix conformations for a significant amount of time, while *ATSP*-7041 is an outlier in this analysis, adopting very stable helical conformations due to the presence of a chemical staple. We used these simulations to define a common reference frame to compare simulations for all peptides in their free and binding simulations (see Methods). Each peptide ensemble was projected onto the corresponding eigenvectors that showed a good separation between helical and non-helical states—as those are the states required for binding (see left panels in Figure 2 and Figure A1, Figure A2, Figure A3 and Figure A4). Clustering on the space defined by the top 14 eigenvectors shows that only *ATSP*-7041 and, to a lesser extent, *pdiq* adopt stable helical structures—consistent with the IDP nature of the other three peptides.

### 2.2. MELD×MD Simulations Balance Exploration and Exploitation of the Binding Energy Landscape

**Figure 3:**
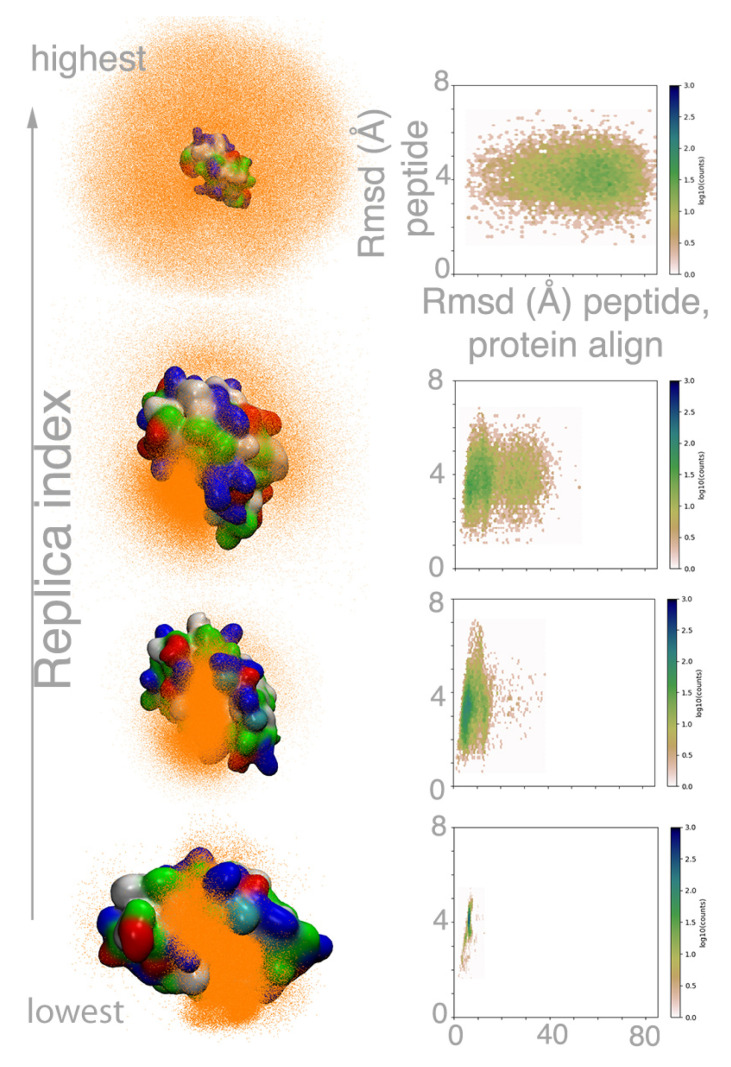
MELD×MD simulations explore unbound states, and different possible binding regions of *p53* on the surface of the protein. The left panel shows a superposition of all peptide conformations (heavy atoms of each conformation are drawn as orange dots) sampled at different replicas. The right panel shows the internal backbone RMSD of the peptide with respect the experimental conformation versus the RMSD of the peptide when aligning to the protein.

**Figure A5:**
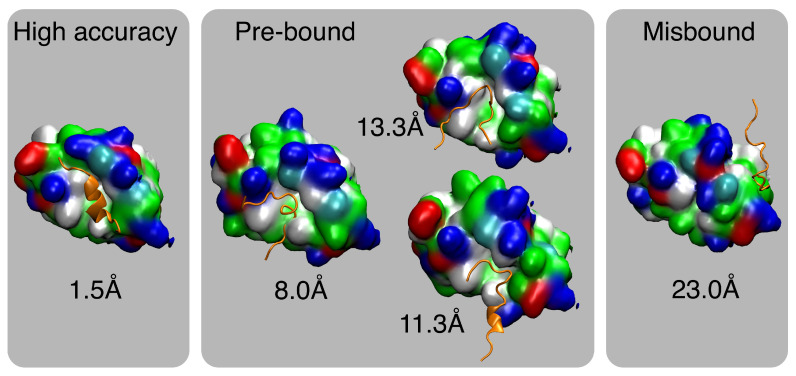
Clustering MELD×MD ensembles correctly identifies the MDM2 pocket as the binding site. Clustering is done by aligning on the MDM2 protein and using that alignment and the RMSD of the peptide between conformations as a distance metric. We define high accuracy binding (left) as the peptide binding in the right pocket with the right conformation (RMSD < 5 Å). For many clusters, the peptide is at least partially occupying the experimental binding site, with incorrect peptide conformations. Our approach samples these conformations at higher replica index, with a few progressing to the experimentally bound conformation. Finally, we find some conformations interacting with MDM2 at different sites in the protein (right panel).

Figure 3 provides a visual outlook on the binding process explored by the MELD×MD replica exchange procedure in terms of the relative position of the peptide with respect the protein and the peptide’s intrinsic conformational preferences. At high replica indexes, the force constants for the restraints are set to zero and the temperature is high (see methods). In these conditions, the peptide samples conformations far away from the active site, distributed uniformly around the protein. During the binding process, the MDM2 flexibility allows for the opening and closing of the binding cavity (see right panel in Figure A5). As the replica index decreases, the temperature decreases and the biasing restraints towards the protein become active, producing a frustrated energy landscape. Under these conditions, the peptide samples conformations on the surface of the protein, identifying early on the MDM2 hydrophobic pocket as the most likely region for binding. Sampling is concentrated in the binding pocket at the lowest replica. Thus, at the highest replica, the protocol favors full exploration of the energy landscape, while, at the lowest replica, it favors full exploitation by sampling around a particular binding region near the protein. The nature of MELD×MD enhances binding/unbinding events by allowing replicas to explore different Hamiltonian and temperature conditions, leading to a different balance of exploration and exploitation [27,28].

### 2.3. Peptides Become More Structured in Proximity to MDM2

**Figure 4:**
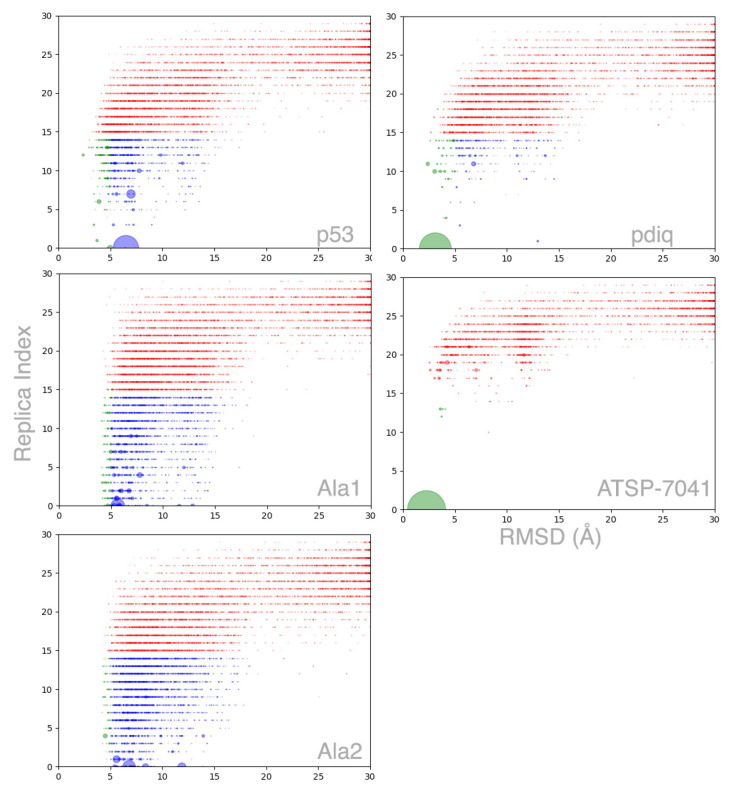
Funneling binding plot for the five peptides. Each dot corresponds to a cluster center from a 2D-RMSD based on all replicas. The larger the circle the larger the population of the cluster. Each circle is plotted at the average RMSD inside that cluster with respect to the native conformation and the mode of the index replica in that cluster. The color code is green (RMSD<5) or blue (RMSD>5) when the mode of the replica index is lower than 15, and red otherwise.

**Figure A6:**
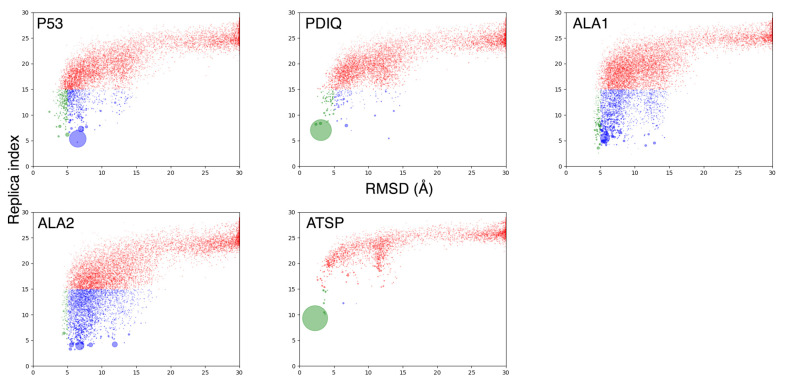
Funneling binding plot for the five peptides. Each dot corresponds to a cluster center from a 2D-RMSD based on all replicas. The larger the circle the larger the population of the cluster. Each circle is plotted at the average RMSD inside that cluster with respect to the native conformation and the average of the index replica in that cluster. The color code is green (RMSD<5) or blue (RMSD>5) when the average of the replica index is lower than 15, and red otherwise.

MELD×MD binding simulations show a higher fraction of helical conformations for all peptides with respect to their free simulations (see middle column in Table 2). However, the increase in helical content for the peptide is not always associated with binding at the correct binding site (right column in Table 2). A 2D-RMSD clustering calculation on all replicas (see Figure 4 and Figure A6) reveals the funneled nature of binding for three peptides. We can identify three broadly defined regions in the funneling plots based on the RMSD distribution: between 0–5 Å (high accuracy binding), 5–15 Å (pre-bound), ∼15–30 Å (misbound), and a fourth region for unbound conformations sampled by higher replicas (see Figure A5). All five peptides identify the binding pocket as the binding site, but the two control sequences bind through multiple backbone conformations with little structural preference. *ATSP*-7041 exhibits the most funneled behavior, rapidly converging onto a large high accuracy native-like cluster. Both *pdiq* and *p53* exhibit a similar behavior, in which all three regions are explored even at the lower replicas, with funneling to one major state. For *p53*, the native configuration is sampled, but is not identified as the most populated cluster. The observed binding mode introduces a kink in the backbone between the helical and non-helical region that is not observed in the experimental structure. The control *Ala1* sequence contains the three anchoring residues present in *p53*, but exhibits a binding profile more similar to control *Ala2*, which lacks the anchoring residues. Thus, the control sequences show that the MELD×MD setup is not over-constraining the peptide to bind in the binding pocket or in the binding conformation, and large cluster populations are reflective of significant binding.

We compare all peptide binding ensembles on equal footing by projecting them on the same eigenvector space as the free peptides. Figure 2 compares the free peptide ensemble with those produced from MELD×MD at the lowest/highest replica index (bound/unbound) for *ATSP*-7041. The figure also shows the clusters arising from the free peptide ensemble, as well as the highest population clusters, from the binding simulations. The preferred conformation for *ATSP*-7041 in its free peptide is the same conformation needed for binding, resulting in significant binding observed throughout the simulations. A similar behavior is observed for *pdiq*, where the ensemble of the free peptide is larger due to the absence of the chemical staple (see Figure A1). For the three IDP peptides (see Figure A2, Figure A3 and Figure A4), the ensembles are even broader than for *pdiq* resulting in a larger number of clusters. The free peptide clusters for these IDP peptides are low in population and lack agreement with the preferred binding mode. However, in binding simulations at low temperature, *p53* explores a narrow conformational ensemble similar to *pdiq* and very different from the broader ensembles sampled by the control sequences. For the two control sequences, the minima of the free ensemble distribution is displaced with respect to the three other peptides, disfavoring bound-like conformations, which result in broader ensembles for the two control peptides during MELD×MD binding simulations.

**Figure A7:**
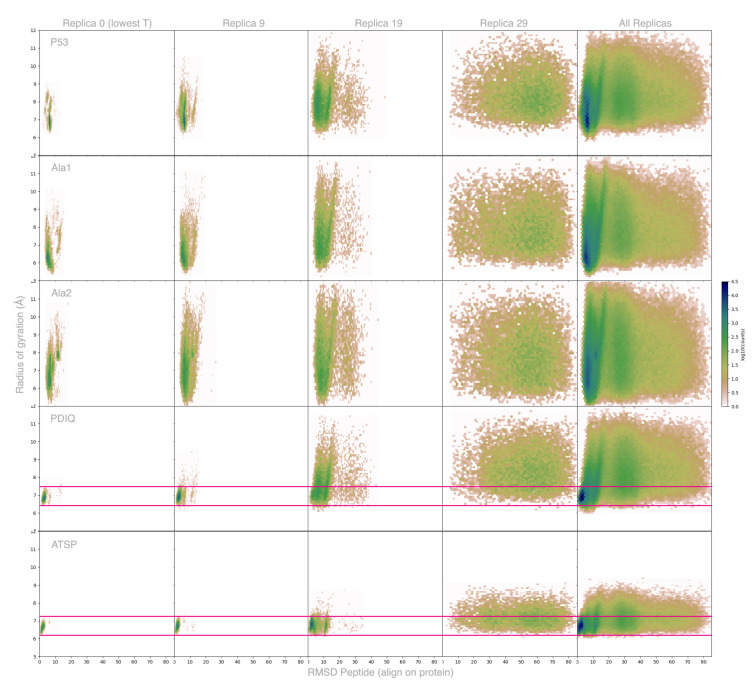
Radius of gyration versus RMSD of the peptide (protein align) for different replicas in MELD×MD for all peptides studied. The red lines for peptides *pdiq* and *ATSP*-7041 depict the region of the radius of gyration explored at the lowest temperature replica.

Complementary knowledge for the binding process emerges from looking at the internal structure of the peptide (radius of gyration) with respect to the position of the peptide to MDM2 (RMSD, see right column in Figure 3 and Figure A7). At high replica index, all peptides sample conformations far from the protein, with large fluctuations in the radius of gyration (between 5 and 12 Å except for the *ATSP*-7041 peptide, where the chemical staple prevents conformations with a radius of gyration above 9 Å). When binding in the MDM2 hydrophobic pocket, the peptide adopts compact conformations with a radius of gyration around 7 Å. This happens early in the binding process (higher replica index) for the *pdiq* and *ATSP*-7041 peptides and is not observed for the *Ala2* control due to the lack of anchoring residues.

### 2.4. Helical Propensities Show Different Binding Patterns

**Figure A8:**
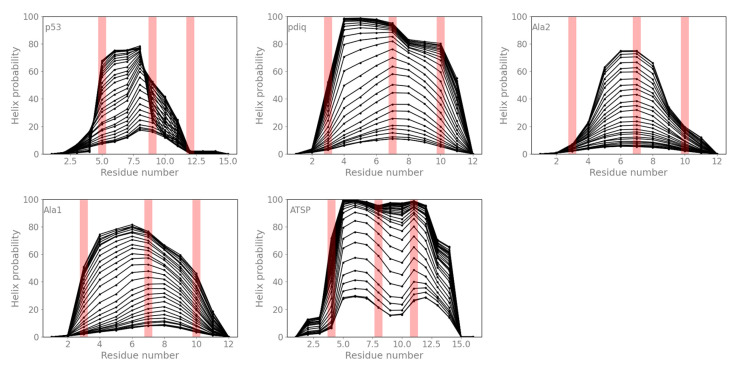
Secondary structure profiles for each peptide in MELD×MD runs. Each line represents a different replica (different H and T). Replicas with higher percentage of secondary structure are sampling at low temperature and ambiguous restraints guiding the peptide to the protein at full strength. Red bars represent the location of the three anchoring residues for each peptide.

The binding ensembles produce a higher helical content with respect to the free-peptide simulations. Figure A8 shows the three anchoring residues to be predominantly in helical conformations for *pdiq* and *ATSP*-7041. *p53* is well known to make a helix in the N-terminal region of the peptide which our simulations reproduce. The *Ala1* control sequence, which has three anchoring residues, is also able to adopt helical conformations, to a lesser extent. The spacing of the anchoring residues in the sequence (residues *i*, i+4 and i+7) and the size of the binding site favor helical conformations for the simultaneous interaction inside the active site. However, the *Ala2* control, adopts very small amounts of helical conformations for two of the three anchoring regions, consistent with the lack of anchoring residues to stabilize those conformations. Not surprisingly, the peptides with larger helical content also have narrower conformational ensembles at low replica index (*ATSP*-7041 and *pdiq*), whereas *p53*, *Ala1*, and *Ala2* have progressively larger ensembles at the lowest temperature replica.

### 2.5. MDM2 Exhibits a sMall Conformational Change upon Binding

**Figure A9:**
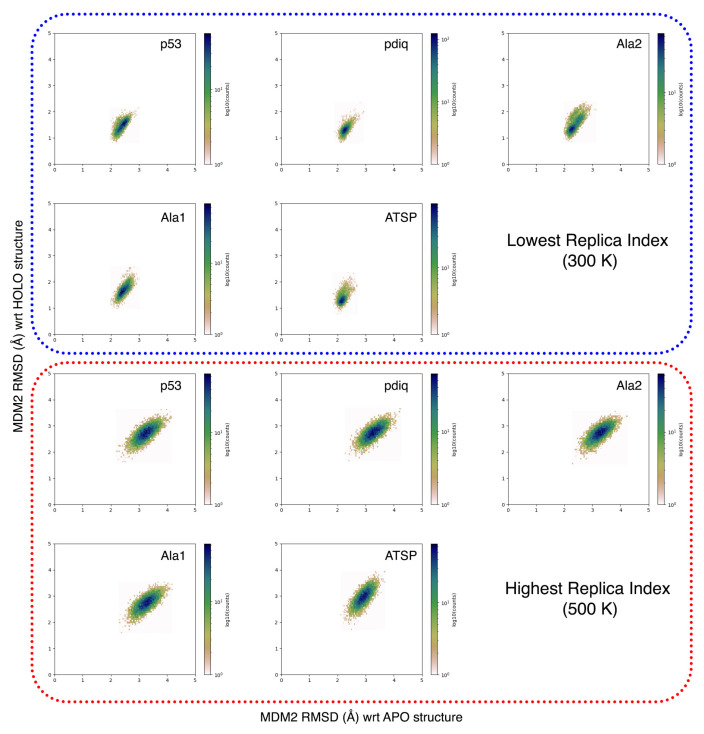
MDM2 protein ensembles with respect to holo and apo experimental structures at the lowest and highest index replicas. Simulations used a flat-bottom harmonic restraint on C${}_{\alpha }$ positions with a 3.5 flat-bottom region.

There is a small conformational rearrangement of the backbone (1.9 Å RMSD) between the apo (unbound) and holo (bound) crystal structures (1z1m [29] and 1ycr [30], respectively), which opens up the cavity for binding. Sidechain rearrangement of the surface residues happens on a faster timescale, changing the surface accessibility to the binding cavity. In MELD×MD binding, the conformational freedom of MDM2 by using flat-bottom harmomic restraints on the $C_{\alpha }$ around the holo structure to prevent unfolding in the replica exchange ladder (see methods). At high temperatures, we find that the protein is sampling conformations between 2–3.5 Åbackbone RMSD from the holo structure and a similar range (2.5–4.0 Å) with respect to the apo MDM2 structure (see Figure A9). At low temperatures, the thermal ensemble is narrower, with an RMSD in between 1 to 2 Åfrom the holo structure and 2–4 Å from the apo structure. The presence of the peptide binding for a significant amount of time to the active site further shifts the RMSD to lower values (see *pdiq* and *ATSP*-7041 in Figure A9). There are no restraints on the sidechains, in which fluctuations determine the open/closed state of the cavity. Reorientation of these sidechains is fast and adapts to the presence of the peptide near the active site.

## 3. Discussion

The debate between conformational selection and induced fit mechanisms of binding is being reconciled into a mixture of the two [7], with different balance of each depending of the system. In the MDM2/p53 system, the protein undergoes a small conformational change from its apo (unbound) to holo (bound) structure, whereas the IDP peptide folds upon binding to the active site. MD simulations of the free *p53* sequence shows its intrinsically disordered nature, with little propensity for helical conformations. Querying the *p53* binding motif in the PDB returns twelve structures, covering four different protein targets: MDM2 [30], MDMX [20], p300 [31], and the CREB-binding protein [32]. In all cases, the *p53* N-terminal domain adopts a helical conformation, but with different sidechain rotameric states [32]. Our binding simulations reproduce these trend, with the size of the conformational ensemble considerably shrinking upon binding (see Figure A2 and Figure A7). Our MELD×MD simulations lose the kinetic information of binding, but, following a particular replica as it goes up and down the replica ladder, we can observe the series of events that lead to binding. In general, we observe a higher helical content for *p53* near the protein surface, consistent with excluded volume effects [33]. The spacing of the anchoring residues (*i*, i+4 and i+7), combined with the size of the hydrophobic pocket favors binding in helical conformations. The funneling towards the binding site driven by hydrophobic interactions in MELD responds to our knowledge that the hydrophobic anchoring residues were vital for binding. Hydrophobic residues on the surface of the protein are more likely in the active site, hence accelerating binding towards it. A different choice of information (e.g., using polar residues) would have resulted in less directed sampling, as polar and charged residues are frequent in the protein surface. Visual inspection shows binding through different modes, with one of the hydrophobic residues anchoring in the cavity and driving the rest; although the peptide does not bind as a helix, it quickly adopts a partial helical structure (see Figure A8) consistent with experiments. The tryptophan seems to be critical for adopting the correct experimental binding conformations: we observe many instances of the peptide bound in the cavity as a helix with the bulky tryptophan interacting with MDM2 hydrophobic sidechains not in the binding pocket, leading to kinks in the backbone structure (see cluster 1 in Figure A2). These incorrect bindings have a long life time in our simulations and require at least partial unbinding before correctly binding according to the experimental motif, which, in our simulations, is sampled but is not the predominant state.

The *pdiq* inhibitor binds experimentally with longer helical motifs covering all amino acids. Analyzing PDB codes 1ycr and 3jzs reveals differences in the secondary structure (using STRIDE [34] as incorporated in VMD (Visual Molecular Dynamics) [35]). The difference in helicity affects the last anchoring residue (leucine), which is in a coil rotameric state for *p53* and in a helical state for *pdiq*. Our simulations show that *pdiq* forms a significant amount of helix in its free form, which favors binding significantly in our binding simulations (see Figure A1 and Figure A8). For *pdiq*, we observe pre-binding in helical conformations, shifted with respect to the experimental binding site, and fast rearrangement of the peptide, sometimes involving rapid helix unfolding and refolding in the binding site leading to the experimental structure. The helical behavior is further accentuated for the *ATSP*-7041 inhibitor, where all binding takes place through helical conformations thanks to the chemical staple. Rearrangements in the active site involve displacements of the alpha helix to allow better interaction of the alpha helices; this is done through partial unbinding of the helix without loosing the helical character. Both control sequences access the binding site unfolded and explore many possible conformations. *Ala1* can sample the helical conformations which favor strong binding as seen from the top clusters (see Figure A3), but they have significantly lower population than the other three binding peptides (see Figure 4). On the contrary, *Ala2* has no anchoring residues and is rarely observed adopting helical conformations in the binding site (see Figure A4). These observations are supported by looking at the narrow conformational binding ensembles sampled at the lowest temperature replica for the peptides that bind (see Figure 2, Figure A1, and Figure A2), in contrast with the broader ensembles of the control peptides (see Figure A3 and Figure A4). Taken together, the results show that the anchoring residues are necessary to adopt the helical conformations associated with good binding to MDM2 but not enough on their own to promote this helical state.

**Figure A10:**
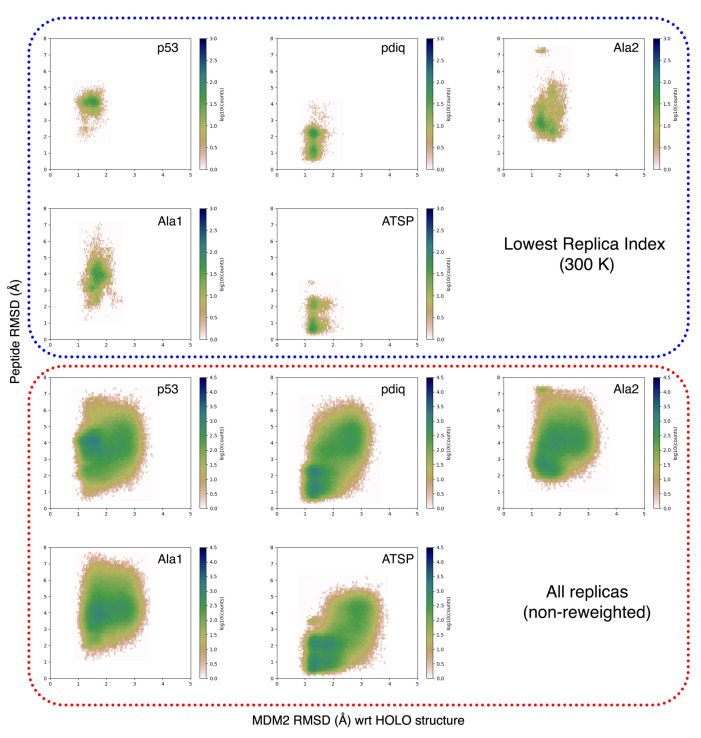
MDM2 versus peptide RMSD ensembles for the five peptides.

The ensembles at different replica index depict the nature of the binding/unbinding dynamics. The control peptides rapidly lose any memory of the bound conformation as the replica index increases (see Figure A7 and Figure A10). For the other three peptides, we observe a bimodal distribution of states: for *p53* centered at 2/4 Å and at 1/2 Å for *pdiq* and *ATSP*-7041. As we increase the temperature in the replica ladder, the higher RMSD state becomes more prevalent. By replica 20, all peptides are sampling broad distributions with very low populations of the 1 RMSD state for the peptide, except for *ATSP*-7041, which, due to the chemical staple, even at high temperatures can sample conformations close to the bound conformation. However, at the highest replica, all peptides have lost memory of the bound conformation. Overall, we can distinguish three regions: an unbound conformation in which the peptide explores conformations far from its bound conformation, a pre-bound conformation, and a bound conformation. Both pre-bound and bound conformation lock the protein close to its holo conformation. For the poly-ala peptide, the bound state is rarely seen, while, for the poly-ala with binding side-chains, it is easier but not the predominant state. The pre-bound state for *pdiq* and *ATSP*-7041 is very close to the bound conformations, while, for *p53*, it is further away.

The binding free energy can be separated into a contribution coming from the conformational preferences of the peptide and protein systems, as well as an interaction contribution ($\Delta G_{bind}=\Delta G_{conformation}+\Delta G_{interaction}$), where $\Delta G_{conformation}$ can further be separated into the protein and peptide contributions ($\Delta G_{conformation}=\Delta G_{conformation}^{peptide}+\Delta G_{conformation}^{protein}$). $\Delta G_{interaction}$ is dictated by the specific interactions between the protein and peptide, which, in this case, arises from the three anchoring residues highlighted in Table 1, as shown by alanine scan mutagenesis studies [25]. Given a force field, MELD×MD samples multiple binding/unbinding events, effectively capturing both free energy contributions, even if it cannot decompose the contribution of each. Using the same protocol for all peptides allows us to identify differences in their binding preferences and peptide conformations. The main advantage is that, in this process, the peptide is completely flexible, free to adopt conformations in response to the environment. We observe the active site changing in response to the presence—and conformation—of the peptide.

**Figure A11:**
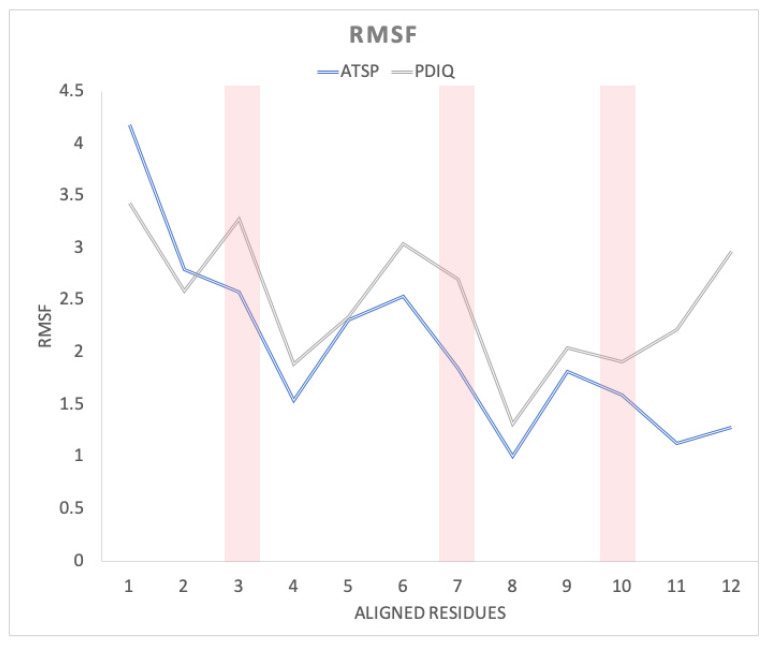
RMSF (Root Mean Square Fluctuation)comparison of the two best peptide binders. The red bars indicate the locations of the anchoring residues. Both peptides have been aligned to match the anchoring residues and end residues removed from *ATSP*-7041 for this analysis.

Our studies hint that the binding mechanisms for *pdiq* and *ATSP*-7041 both favor initial binding as helices, with different mechanisms for rearrangement. Since kinetics are lost in our replica exchange ladder, testing this hypothesis will require future work in which the states discovered from our ensembles can be used for seeding unbiased simulations to construct markov models that show the binding pathways [36,37,38]. The chemical staple successfully increases the helical content, but it also plays a role in reducing side chain rotamer freedom through the steric volume it occupies (see Figure A11). Thus, *ATSP*-7041 is predisposed to make helical conformations, and to establish the right interactions. Figure 2 shows only two clusters: a major cluster binding as a helix with the three anchoring residues in the active site and a minor one with the staple in the active site. For *pdiq*, we see a higher number of minor clusters (see Figure A1) exhibiting helical conformations, in which at least one anchoring residue is not in the active site.

Thus, for accurate modeling of the *p53*-MDM2 interaction, we need to capture: (1) the intrinsic peptide propensity to helical conformations and (2) type and alignment of the anchoring residues inside the binding cavity. Peptides that, in their free form, favor helices seem to favor binding (given the same interface residues) by reducing the $\Delta G_{conformation}^{peptide}$. However, even when shifting the helical propensities, binding simulations are needed as the binding mode can change (as we see for *ATSP*-7041 and *pdiq*).

## 4. Materials and Methods

### 4.1. Choice of Peptide Systems

We chose a set of five peptides for this study: the sequence from the *p53* binding epitope, two high affinity inhibitors (*pdiq* [39] and *ATSP*-7041) [25] and two control peptides, based on the poly-Ala sequence (*Ala1* and *Ala2*; see Table 1). Of the two control sequences, *Ala1* sequence conserves the set of hydrophobic residues that allow binding, and *Ala2* does not. *ATSP*-7041 is a stapled peptide using three non-standard amino acids, where one of the three anchoring residues (Leucine) is substituted by a non-canonical amino acid.

For *p53* and *pdiq*, we used crystal structures of the peptides binding to MDM2 (PDB codes 1ycr [30] and 3jzs [39]. For *ATSP*-7041, we used the structure bound to MDMX (PDB code 4n5t [25]) and superposed the active site onto MDM2 to have the reference structure of the peptide on the active site of MDM2. For the two control peptides bases on poly-ALA, there is no native structure. We compare it to the *p53*-MDM2 conformation for those two peptides. Parameters for the *ATSP*-7041 peptide are derived from the general amber force field (GAFF) [40], deriving charges based on the AM1 model [41].

### 4.2. Free Peptide Simulations

We used the ff14SB force field for amino acid sidechains [42] and the ff99SB force field for backbone parameters [43], using the GBneck2 implicit solvent model (igb = 8) [44] to improve sampling efficiency. We ran the simulations for 2 μs using hydrogen mass repartitioning [45] with a 4fs timestep using the Amber molecular dynamics package [46]. A concern with implicit solvents is the bias towards some secondary structure [47]. However, this combination of force field with implicit solvent has shown to be reliable in reproducing the folding of peptide and protein systems [44,48,49].

### 4.3. MELD×MD Binding Simulations

We ran 1 μs-long H,T-REMD simulations using OpenMM [50] with the MELD plugin [26]. MELD allows us to incorporate noisy information to increase the sampling in regions of interest [51,52]. In this case, our interest was in observing the peptide-protein association. We required that there were at least five heavy-atom contacts between the three anchoring residues in the peptide (F, W, and L in *p53*) and any other hydrophobic residue in MDM2, andthe pool of possible contacts was selected from the combinatorics of both sets. The restraints were imposed using flat-bottom harmonic restraints. The flat region was defined as a pair of residues closer than 5 from each other, the restraints increased quadratically up to 7 and linearly beyond, with a force constant of 250 J/K/mol. At every timestep, all possible restraints are evaluated, sorted by energy, and only the lowest 5 in restraint energy are used until the next timestep. In this way, no information is lost as the simulation progresses.

The H,T-REMD protocol includes 30 replicas, where the change in Hamiltonian affects the force constant of the restraints. The 30 replicas are mapped to a value of alpha (α) between 0 (lowest replica) and 1 (highest replica). The Hamiltonian and temperature have defined values of the restraint force constant and temperature as a function of alpha. The temperature increases geometrically from 300 K (α=0) to 500 K (α=0.5) and is kept at this temperature for higher values of alpha. The force constants for the restraints is set to 0 J/K/nm${}^{2}$ at α=1 and is gradually increased to the value of 250 J/K/nm${}^{2}$ for α≤0.6. Exchanges between active restraints are more likely at higher index replicas.

### 4.4. Clustering Analysis

We use 2D-RMSD hierarchical clustering using a single linkage scheme within cpptraj [53] and report the centroid structure of each cluster and its population as representative of the clusters. We used the last 500ns of each replica, aligning on the protein ($C_{\alpha }$ and $C_{\beta }$ atoms) and clustering on the overlapping peptide residues ($C_{\alpha }$ and $C_{\beta }$ atoms). For Figure 2, the lowest temperature replica and all replicas for the clustering of the funnel plots, both with ϵ =1.5. For those in Figure 2 and Figure A1, Figure A2, Figure A3 and Figure A4, we increase ϵ to 2.0, to depict more diverse clusters.

### 4.5. Projections Onto a Common Feature Space

We used pyEMMA [54] to featurize our system according to phi and psi dihedrals by choosing a common set of residues on all peptide systems resulting in 22 dihedrals, and we used dihedral shifting to reduce discontinuities in the distibution rather than using sine and cosines on the dihedrals [55]. The ensembles from free *p53*, *pdiq*, and *Ala1* were chosen as a common ensemble before dimensionality reduction of the system by using time-independent coordinate analysis [56] with a lag time of 10 ns, from which we extracted the top 14 eigenvectors that account for 95% of the variance. We then projected each peptide ensemble (from free and bound simulations) into the top eigenvectors. Finally, we performed clustering of the free peptide ensembles in the space defined by the top 14 eigenvectors to produce Figure 2 and Figure A1, Figure A2, Figure A3 and Figure A4. Since the vectors were calculated for intrinsically disordered ensembles of the free form of the peptides, they are not representative of the slowest transitions during the binding process, which we cannot extract from the MELD-biased ensembles. Nonetheless, they provide a common set of vectors to represent all free and bound peptide systems studied. For these plots, we decided to project onto the third and fifth eigenvectors since these offered the best separation between clusters for the relevant states during binding.

## 5. Conclusions

Predicting bound structures for IDP peptides that fold upon binding is a computational grand challenge. We have shown that possible peptide inhibitors do not necessarily bind with the same binding mode, requiring modeling approaches that allow identification of the correct binding pose. The method successfully reproduces the binding of the two inhibitors and the *p53* epitope, while showing that the two control peptides are unsuccessful binders. We further show that, by changing the intrinsic properties (e.g., helical propensity, in this case), we can identify better binders; this simplifies the design of peptide inhibitors into two distinct tasks: optimizing interface residues and optimize structural propensities. The first task requires knowing the binding mode, and the second one can be assessed by MD simulations on the free peptide, at a lower computational cost than the binding simulations. Finally, we have shown that MELD×MD is a useful tool to handle flexible binding and helps to ensure that the designed binders indeed bind and what their preferred binding mode is.

## Figures and Tables

**Table 1:** Peptides used in the current work. Bold letters represent the anchoring residues.

Name	Sequence																
*p53*	S	Q	E	T	**F**	S	D	L	**W**	K	L	**L**	P	E	N	
*pdiq*			E	T	**F**	E	H	W	**W**	S	Q	**L**	L	S	
*Ala1*			A	A	**F**	A	A	A	**W**	A	A	**L**	A	A	
*Ala2*			A	A	A	A	A	A	A	A	A	A	A	A	
*ATSP*-7041		ACE	L	T	**F**	R8	E	Y	**W**	A	Q	**Cba**	S5	S	A	A	NHE

**Table 2:** Populations for peptides in free and MELD binding simulations. Clustering is done on the lowest temperature replica using hierarchical clustering with ϵ=1.5.

Name	Peptide Population (% Top Cluster)		
	**Unrestrained MD**	**MELD×MD** **(Peptide Align)**	**MELD×MD** **(Protein Align)**
*p53*	0.6	70.6	46.1
*pdiq*	24.0	97.6	95.3
*Ala1*	1.4	54.7	16.0
*Ala2*	0.2	31.3	17.5
*ATSP*-7041	69.5	97.8	91.6

## Data Availability

The MELD code used to run binding simulations is available to download from github: https://github.com/maccallumlab/meld.
